# FOXF2 inhibits proliferation, migration, and invasion of Hela cells by regulating Wnt signaling pathway

**DOI:** 10.1042/BSR20180747

**Published:** 2018-10-17

**Authors:** Jun Zhang, Chunxia Zhang, Lin Sang, Ling Huang, Juan Du, Xingbo Zhao

**Affiliations:** 1 Department of Obstetrics and Gynecology, Shandong Provincial Hospital Affiliated to Shandong University, No. 324, Jing wu wei qi Road, Ji’nan City 250021, Shandong Province, China; 2 Department VI of Obstetrics, Tai’an City Central Hospital, No. 29, Long tan Road, Tai’an City 271000, Shandong Province, China; 3 Department of Obstetrics and Gynecology, The Second People’s Hospital of Hefei City Affiliated to Anhui Medical University, Guang de Road, Yao hai District, Hefei City 230000, Anhui Province, China

**Keywords:** Cervical cancer, FOXF2, Invasion, Proliferation, Wnt signaling pathway

## Abstract

This article was aimed to study the FOXF2 effects on cervical cancer. Tumor tissues and adjacent tissues of 41 cervical cancer patients were collected. Human endometrial epithelial cells (hEEC) and Hela cells were cultured. FOXF2 expression vector and its empty vector were transfected into Hela cells, and named as pcDNA 3.1-FOXF2 group and Vector group, respectively. Hela cells without any treatment were set as Blank group. qRT-PCR was used to detect mRNA expression. Nude mouse xenograft assay was performed to test Hela cells proliferation ability *in vivo*. FOXF2 and β-catenin positive cell numbers were detected by immunohistochemistry. Protein expression was analyzed by Western blot. Cells migration and invasion were conducted by Transwell. Tumor tissues and Hela cells FOXF2 expression were lower than that in adjacent tissues and hEEC (*P*<0.01). Low FOXF2 expression predicted poor outcomes of cervical cancer patients. Compared with Blank group and Vector group, Hela cells of pcDNA 3.1-FOXF2 group were with higher FOXF2 expression, lower OD_495_ value, migrated and invaded cells, higher E-cadherin expression, lower Vimentin and Snail expression, smaller tumor volume in nude mice, lower c-Myc, CyclinDl, MMP9, Lgr5, and nuclear β-catenin expression (all *P*<0.01). FOXF2 inhibits Hela cells proliferation, migration, and invasion through regulating Wnt signaling pathway.

## Introduction

Cervical cancer is the third most common tumor amongst women in the world. Researchers revealed that in United States, ~12000 patients were diagnosed with cervical cancer each year [1] and 4100 cases died [2]. Despite the fact that a slight decline in cervical cancer incidence was found over the past decade, cervical cancer is still the second leading cause of cancer-related deaths amongst women in developing countries [3]. Consistent with other malignancies, it was difficult to fundamentally cure cervical cancer through traditional treatment, such as surgical resection, radiotherapy, and chemotherapy [4,5]. With the development of medicine, many researchers considered that treatment of tumors at the genetic level could achieve the goal of complete cure. Therefore, the discovery of effective therapeutic targets is very important for the advancement of cancer treatment.

FOXF2 was found to be associated with the development of multiple tumors. Kong et al. [6] demonstrated that FOXF2 was a new independent predictive factor of non-small cell lung cancer. Its lower expression could lead to poor prognosis of patients, especially for patients with stage I non-small cell lung cancer. They also revealed in their another article that down-regulation of FOXF2 was a sign of early-onset metastasis and poor prognosis of patients with breast cancer [7]. Wang et al. [8] identified in their research that FOXF2 acted as a novel epithelial–mesenchymal transition (EMT) suppressing transcription factor in basal-like breast cancer. Their further research also showed that FOXF2 promoted basal-like breast cancer cells metastasis by up-regulation of TWIST1 as well as activating EMT. Dysregulation of FOXF2 was also linked to many other cancers, such as prostate cancer, esophageal cancer, and colorectal cancer [9–11].

In the present study, we researched the expression level of FOXF2 in cervical cancer and its effect on cervical cancer cells proliferation, migration, and invasion. As far as we know, there were rarely literatures to report the relationship between FOXF2 expression and cervical cancer. This research will provide new potential therapeutic targets for the treatment of cervical cancer.

## Materials and methods

### Sample collection of cervical cancer patients

The patients who were admitted to our hospital and diagnosed with cervical cancer from 2016 to 2017 were enrolled in the present study. The patients who had been diagnosed with cervical cancer for the first time and had never used hormone therapy and radiochemotherapy were included. While those with other severe organic lesions or neurological or mental disorders were excluded. At last, 41 patients meeting the above criteria were included in the study, and their tumor tissues and adjacent tissues were collected during surgery. The average age of these patients was 55.67 ± 8.21 years. The detailed clinical characteristics of patients were shown in Table 1. All patients have signed informed consent and the present study has been approved by the ethics committee of our hospital.

**Table 1:** Demographic characteristics of patients

Demographic characteristics	*n*	FOXF2 protein relative expression	*t* value	*P*-value
Age			−2.632	0.0121
≥50 years	31	0.421 ± 0.0403		
<50 years	10	0.457 ± 0.0268		
Lymph node metastasis			−3.062	0.004
Yes	19	0.411 ± 0.0315		
No	22	0.446 ± 0.0403		
Myometrial invasion			−2.515	0.0161
Yes	25	0.418 ± 0.0364		
No	16	0.448 ± 0.0386		
TNM stage			3.734	0.0006
I–II	27	0.445 ± 0.0321		
III–IV	14	0.402 ± 0.0401		

TNM, Tumor Node Metastasis.

### Cell culture

Human endometrial epithelial cells (hEEC) and cervical cancer Hela cells were cultured in DMEM containing 10% FBS. Both cell lines were purchased from Shanghai Bioleaf Biotech Co., Ltd, China. These cells were individually inoculated into 24-well plates at a density of 1 × 10^5^ cells per well, and incubated in a 5% CO_2_, 37°C incubator.

### Cell transfection and grouping

FOXF2 overexpression sequence was inserted into pcDNA 3.1 vector. Hela cells transfected by FOXF2 overexpression vector were set as pcDNA 3.1-FOXF2 group. Furthermore, pcDNA 3.1 empty vector was used to transfect Hela cells, and these cells were named Vector group. Transfection was performed using Lipofectamine 2000 Transfection Kit (Invitrogen, U.S.A.). All transfection sequences were synthesized by Shanghai Jema Pharmaceutical Co., Ltd. In addition, Hela cells without any treatment were set as Blank group. Hela cells of these three groups were dispersed into cell suspensions at a density of 1 × 10^5^/ml by DMEM (10% FBS). Then they were inoculated in 24-well plates, respectively, with 1 ml cell suspensions each well. These 24-well plates were placed in a 5% CO_2_, 37°C incubator for 48 h.

### qRT-PCR detection

Tumor tissues and adjacent tissues were ground in liquid nitrogen. hEEC and Hela cells of each group were also collected. Total RNA in tissues and cells were obtained by using Trizol kits (Invitrogen, U.S.A.). Single-stranded cDNA template was obtained by reverse transcription. Then PCR amplification reaction was performed under the following conditions: 95°C for 30 s; 58°C for 60 s; 72°C for 34 s. Forty cycles were included in this amplification reaction, and 1 μl of cDNA, 1 μl of forward primer, 1 μl of reverse primer were also included. Primer sequence was as follows: FOXF2, forward, TCGCTGGAGCAGAGCTACTT, reverse, CCCATTGAAGTTGAGGACGA; E-cadherin, forward, TGATTCTGCTGCTCTTGCTG, reverse, CTCTTCTCCGCCTCCTTCTT; Vimentin, forward, GAGAACTTTGCCGTTGAAGC, reverse, AAGGTGACGAGCCATTTCC; Snail, forward, TTTACCTTCCAGCAGCCCTA, reverse, GGACAGAGTCCCAGATGAGC; β-catenin, forward, TGCCAAGTGGGTGGTATAGAGG, reverse, CGCTGGGTATCCTGATGTGC; GAPDH, forward, GTCGATGGCTAGTCGTAGCATCGAT, reverse, TGCTAGCTGGCATGCCCGATCGATC. All experiments were performed three times. Data analysis was done by 2^−ΔΔ*C*_t_^ method.

### Transwell assay for cell migration and invasion

Cells were collected and prepared as cell suspension by DMEM (without FBS). Then they were seeded in the upper chamber (with or without Matrigel) of the Transwell chamber. DMEM (10% FBS) was then added into the lower chamber. Incubation at 5% CO_2_, 37°C incubator for 24 h was performed for these cells. After 24 h, Transwell chamber was taken out and residual medium in the upper chamber was gently removed. Cells on the upper chamber were fixed with formaldehyde for 5 min. Crystal Violet was used to dye for 10 min. Then cells on the upper chamber were observed and counted under inverted microscope. Five fields were randomly selected for observation and counting.

### Nude mouse xenograft experiment

Fifteen male nude mice with no statistical difference in body weight (5–6 weeks of age) were reared in a sterile environment for 1 week. They were randomly divided into three groups: Blank group, Vector group, and pcDNA 3.1-FOXF2 group. Five nude mice were included in each group. All nude mice were subjected to skin disinfection. A total of 1 ml cell suspension (1 × 10^4^/ml) was injected into the dorsal side of the right hind limb. Cell suspension injected into each group of nude mice was consistent with cell grouping. After injection, all nude mice were returned to the cages and were continued for 6 weeks under the same conditions. A vernier caliper was used to measure long diameter (a) and short diameter (b) of subcutaneous tumors weekly. Tumor volume was calculated according to the following formula: tumor volume = (a*b^2^)/2. At week 6, all nude mice were killed to remove subcutaneous tumors.

### Immunohistochemical detection

Cervical cancer tumor tissues, adjacent tissues, as well as xenograft tumors in nude mice were routinely paraffin-embedded and sectioned. Ten consecutive slices of each tissue were subjected to xylene dewaxing and gradient alcohol rehydration. Then they were placed in boiling 0.01 M citrate buffer for antigen retrieval. H_2_O_2_ (3%) was added for 15-min incubation at room temperature. Goat serum blocking solution was then added for other 15-min incubation after washing by PBS for three times. Ten sections of each tissue were equally divided into two groups, and 50 μl of rabbit anti-human FOXF2 or β-catenin antibodies (1:100, Santa Cruz Biotechnology) were added respectively for 4°C incubation overnight. PBS was used for three times washing. Secondary antibody was added for 15 min incubation at 37°C. DAB chromogenic reaction and Hematoxylin counterstaining for 30 s were performed sequentially. At last, these slices were sealed with neutral gum after dehydration. Under microscope, five non-overlapped fields of each slice were selected for observation and FOXF2 positive cells were counted. FOXF2 is mainly expressed in the nucleus. Thus, cells that appeared as brown particles in the nucleus were considered to be FOXF2 positive cells.

### Western blot analysis

Tumor tissues and adjacent tissues which were ground in liquid nitrogen were collected. hEEC and Hela cells of each group were also collected. RIPA lysis buffer was used to extract total proteins in these tissues and cells. In addition, nucleus proteins of Hela cells of each group were also obtained through using nucleus protein extraction kit (Boster Biological Technology, Ltd., Wuhan, China). Separation of proteins was conducted by SDS/PAGE at 120 V. Then 2 h blocking with skimmed milk (5%) was performed at room temperature. Primary antibodies used in the present study were rabbit anti-mouse FOXF2, E-cadherin, Vimentin, Snail, β-catenin, c-Myc, CyclinDl, MMP9, and Lgr5, respectively (1:1000, Abcam, U.S.A.). After 12-h incubation with primary antibody at 4°C, three-times washing by TBST were implemented. Subsequently, goat anti-rabbit IgG secondary antibody (1:2000, Beijing Zhongshan Jinqiao Biotechnology Co., Ltd., China) was added to incubate for 1 h at room temperature. TBST was also used for washing three times. GAPDH was set as internal reference of total proteins in cells. Nucleus proteins of Hela cells were normalized to Histone H3.

### Statistical analysis

Data were expressed as mean ± S.D. Comparison between two groups was analyzed by *t* test, and one-way ANOVA test was selected for comparing more than two groups. SPSS 17.0 and GraphPad Prism 5.0 were used for statistical analysis. *P*<0.05 was considered statistically significant.

## Results

### Decreased FOXF2 predicted poor outcomes of cervical cancer patients

**Figure 1:**
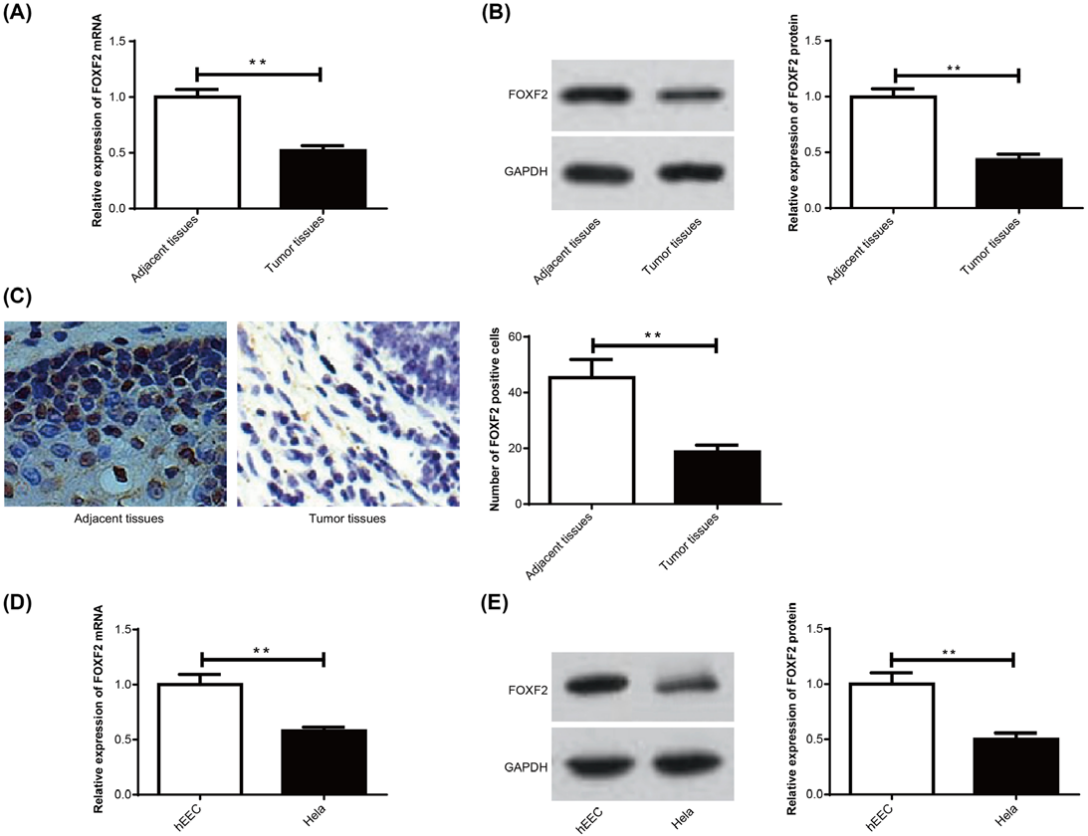
Down-regulation of FOXF2 in cervical cancer tumor tissues and Hela cells (**A**) qRT-PCR detection of *FOXF2* mRNA expression in cervical cancer tumor tissues and adjacent tissues. (**B**) Western blot assay of FOXF2 protein expression in cervical cancer tumor tissues and adjacent tissues. (**C**) Immunohistochemical detection of FOXF2 protein positive cells in cervical cancer tumor tissues and adjacent tissues. (**D**) qRT-PCR detection of *FOXF2* mRNA expression in hEEC and Hela cells. (**E**) Western blot assay of FOXF2 protein expression in hEEC and Hela cells; **P<0.01.

As reported in previous studies, FOXF2 was declined in many kinds of tumors. In this research, we also observed significantly declined *FOXF2* mRNA and protein relative expression in cervical cancer tumor tissues when compared with that in adjacent tissues (*P*<0.01) (Figure 1A,B). Immunohistochemistry also showed that FOXF2 positive cell numbers of cervical cancer tumor tissues were significantly lower than that of adjacent tissues (*P*<0.01) (Figure 1C). In addition, the relationship of FOXF2 protein expression with patients’ demographic characteristics was also analyzed. As shown in Table 1, FOXF2 protein relative expression was significantly associated with age, lymph node metastasis, myometrial invasion, and TNM stage. The patients along with ≥50 years or lymph node metastasis or myometrial invasion or III–IV TNM stage had much lower FOXF2 protein relative expression than the other patients (*P*<0.05 or *P*<0.01). FOXF2 was dramatically down-regulated in cervical cancer patients, and low FOXF2 expression predicted poor outcomes of cervical cancer patients.

Meanwhile, FOXF2 expressions in hEEC and Hela cells were also explored. It could be noticed that, compared with *FOXF2* mRNA and protein expression in hEEC, it was significantly decreased in Hela cells (*P*<0.01) (Figure 1D,E). FOXF2 was also remarkably down-regulated in Hela cells.

### Up-regulation of FOXF2 in Hela cells after transfection

**Figure 2:**
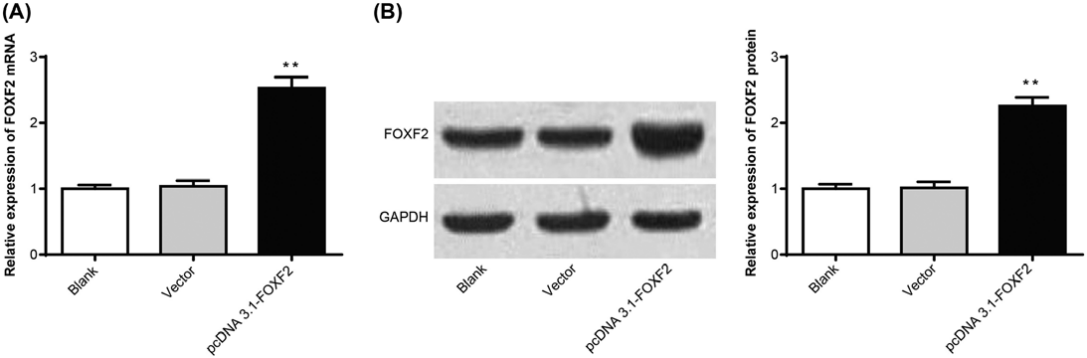
Up-regulation of FOXF2 in Hela cells after transfection (**A**) qRT-PCR detection of *FOXF2* mRNA expression in Hela cells of each group. (**B**) Western blot assay of FOXF2 protein expression in Hela cells of each group. **P<0.01 when compared with Blank group or Vector group.

After transfection, *FOXF2* mRNA and protein relative expression in pcDNA 3.1-FOXF2 group were significantly up-regulated compared with Blank group and Vector group (*P*<0.01) (Figure 2A,B), illustrating that Hela cells were successfully transfected.

### Overexpression of FOXF2 inhibited Hela cells proliferation, migration, and invasion

**Figure 3:**
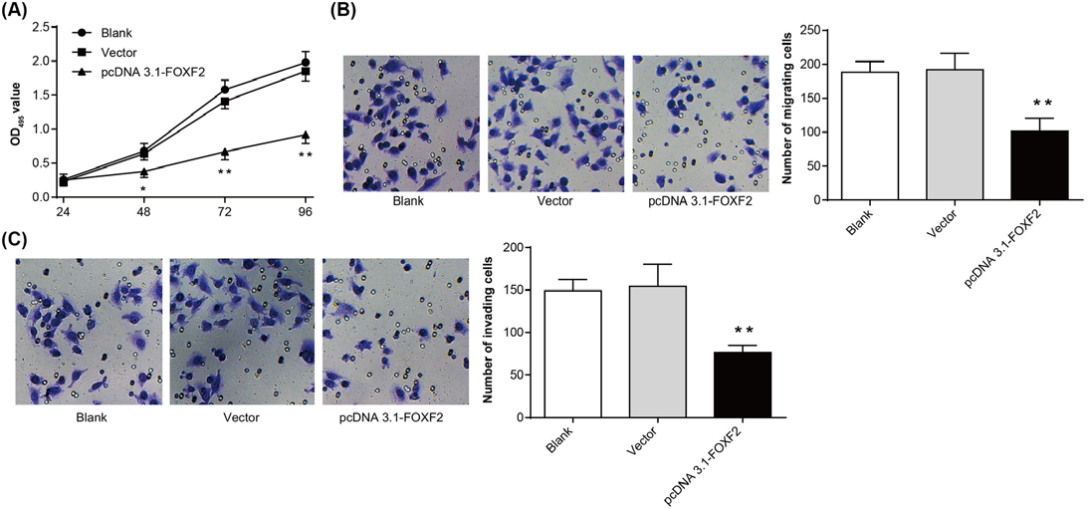
Overexpression of FOXF2 inhibited Hela cells proliferation, migration, and invasion (**A**) MTT assay for Hela cells proliferation in each group. (**B**) Transwell detection of Hela cells migration in each group. (**C**) Transwell detection of Hela cells invasion in each group. *P<0.05 or **P<0.01 when compared with Blank group or Vector group.

MTT assay showed that 48 h after transfection, dramatically lower OD_495_ value was found in pcDNA 3.1-FOXF2 group when compared with Blank group and Vector group (*P*<0.05 or *P*<0.01) (Figure 3A). We also found that the number of migrating and invading cells in pcDNA 3.1-FOXF2 group was 101 ± 16 and 76 ± 7, respectively, which was significantly lower than those of Blank group (188 ± 12 and 149 ± 11) and Vector group (192 ± 20 and 154 ± 21) (*P*<0.01) (Figure 3B,C). All of these results revealed that overexpression of FOXF2 had a significant inhibitory effect on Hela cells proliferation, migration, and invasion.

### Overexpression of FOXF2 affected EMT-related genes expression in Hela cells

**Figure 4:**
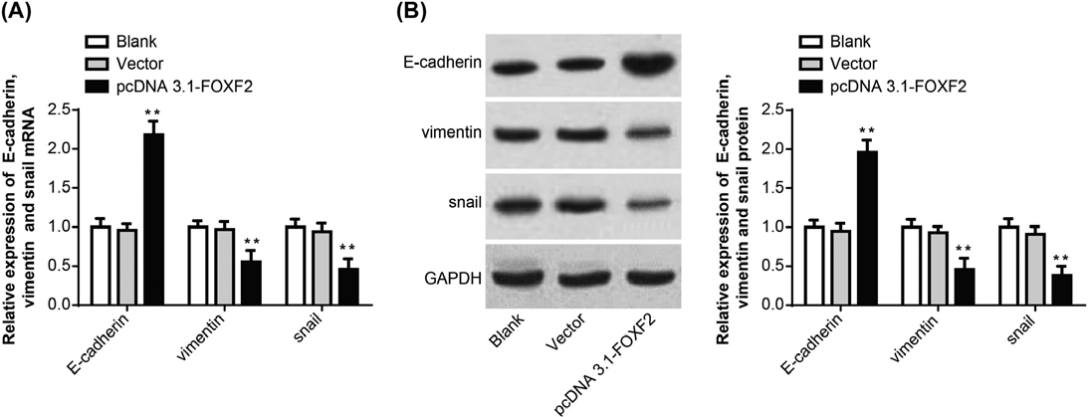
Overexpression of FOXF2 affected EMT-related genes expression in Hela cells (**A**) qRT-PCR detection of E-cadherin, Vimentin, Snail mRNA expression in Hela cells of each group. (**B**) Western blot assay of E-cadherin, Vimentin, Snail protein expression in Hela cells of each group. **P<0.01 when compared with Blank group or Vector group.

There was no statistically significant difference in the relative expression of E-cadherin, Vimentin, Snail mRNA, and protein between Blank group and Vector group. However, when compared with Blank group and Vector group, significantly up-regulated E-cadherin mRNA and protein relative expression as well as significantly down-regulated Vimentin, Snail mRNA, and protein relative expression was found in pcDNA3.1-FOXF2 group (*P*<0.01) (Figure 4A,B). FOXF2 could affect EMT-related genes expression in Hela cells.

### Overexpression of FOXF2 inhibited Hela cells growth in nude mice

**Figure 5:**
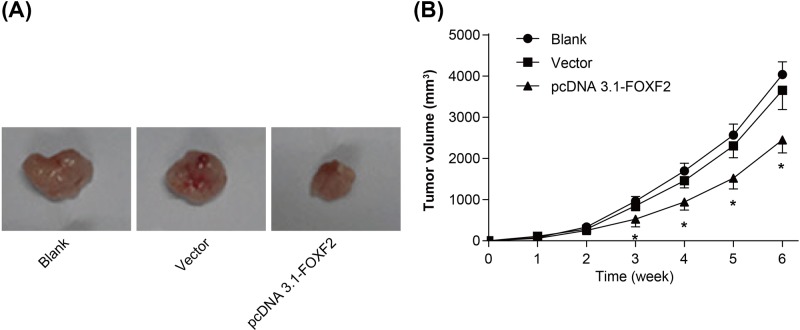
Overexpression of FOXF2 inhibited Hela cells growth in nude mice (**A**) Transplanted tumors of nude mice in each group at 6 weeks after transplantation; (**B**) changes in tumor volume after subcutaneous transplantation in nude mice. *P<0.05 when compared with Blank group or Vector group.

We transplanted Hela cells of each group into nude mice subcutaneously. At 3–6 weeks after transplantation, the subcutaneous tumor volume of pcDNA 3.1-FOXF2 group was markedly lower than that of Blank and Vector groups (*P*<0.05) (Figure 5A,B), illustrating that overexpression of FOXF2 could inhibit Hela cells growth in nude mice.

### Up-regulation of β-catenin in cervical cancer tumor tissues and transplanted tumor tissues of nude mice by FOXF2 overexpression

**Figure 6:**
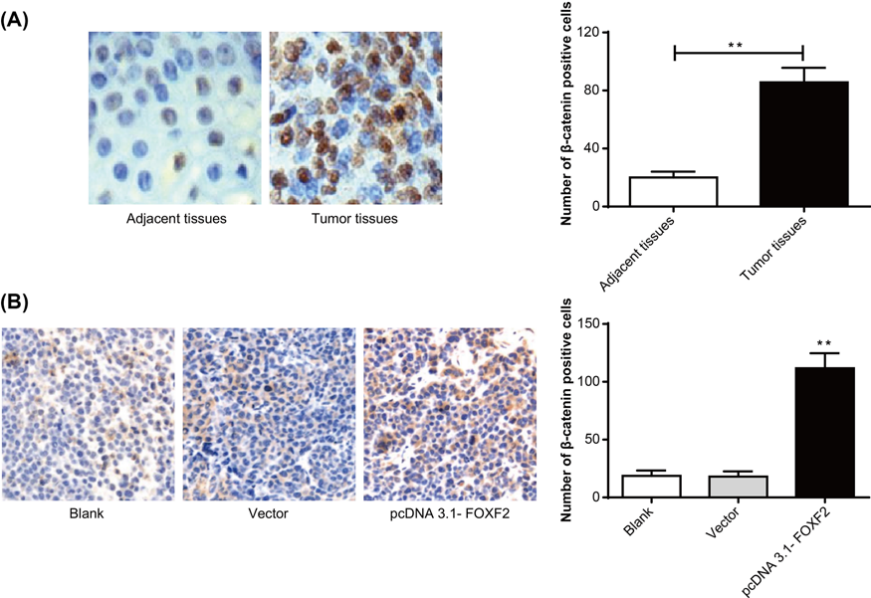
Up-regulation of β-catenin in cervical cancer tumor tissues and transplanted tumor tissues of nude mice by FOXF2 overexpression (**A**) Immunohistochemical detection of β-catenin positive cells in cervical cancer tissues and adjacent tissues; **P<0.01. (**B**) Immunohistochemical detection of β-catenin positive cells in transplanted tumor tissues of nude mice; **P<0.01 when compared with Blank group or Vector group.

According to immunohistochemistry results, we found that the number of β-catenin positive cells in cervical cancer tissues was significantly increased when compared with that of adjacent tissues (*P*<0.01) (Figure 6A). Furthermore, β-catenin positive cell numbers of nude mice transplanted tumor tissues in pcDNA 3.1-FOXF2 group was also higher than that in Blank group and Vector group (*P*<0.01) (Figure 6B). These results indicated that *β-catenin*, a key gene in the Wnt signaling pathway, was dramatically up-regulated in cervical cancer tisssues by FOXF2 overexpression.

### Overexpression of FOXF2 inhibited the expression of target genes in the Wnt/β-catenin signaling pathway and reduced β-catenin expression level in the nucleus

**Figure 7:**
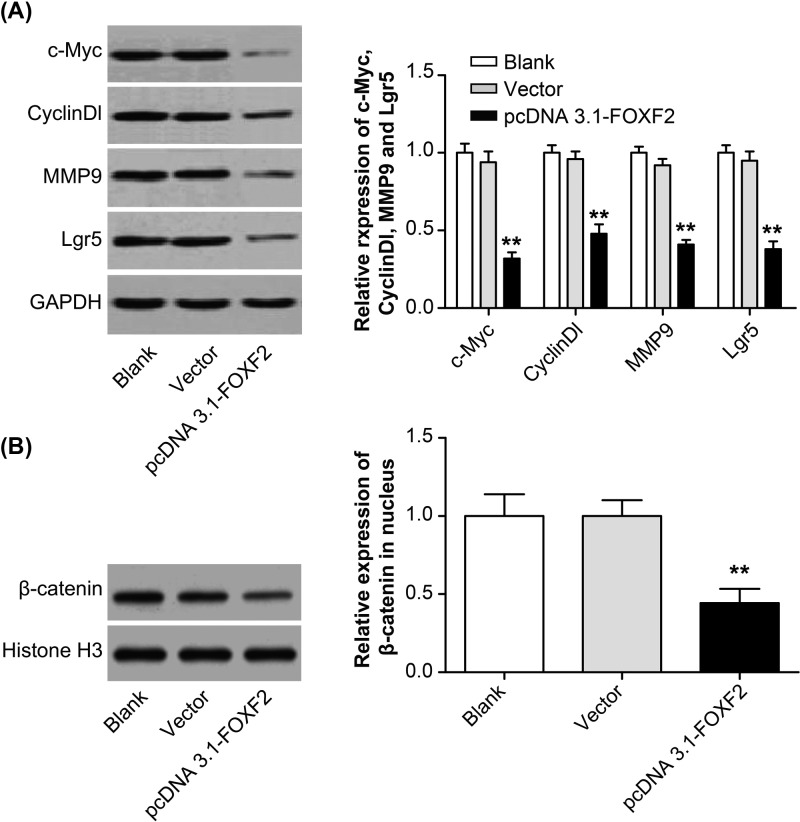
Overexpression of FOXF2 inhibited the expression of target genes in the Wnt/β-catenin signaling pathway and reduced β-catenin *expression level* in the nucleus (**A**) Western blot analysis of c-Myc, CyclinDl, MMP9, and Lgr5 expression in Hela cells of each group. (**B**) Western blot analysis of the nuclear expression of β-catenin in Hela cells. **P<0.01 when compared with Blank group or Vector group.

Expression of target genes (*c-Myc, CyclinDl, MMP9*, and *Lgr5*) in Wnt/β-catenin signaling pathway was detected. Significantly decreased c-Myc, CyclinDl, MMP9, and Lgr5 protein relative expression was found in pcDNA 3.1-FOXF2 group when compared with Blank group and Vector group (*P*<0.01) (Figure 7A), demonstrating that overexpression of FOXF2 inhibited c-Myc, CyclinDl, MMP9, and Lgr5 expression. In addition, our further research also showed that compared with Blank group and Vector group, the relative expression of β-catenin in the nuclei of Hela cells in pcDNA 3.1-FOXF2 group was significantly decreased (*P*<0.01) (Figure 7B). Overexpression of FOXF2 inhibited the expression of target genes in the Wnt/β-catenin signaling pathway and reduced β-catenin expression level in the nucleus.

## Discussion

FOXF2 was an important member of FOX family, which regulated the promoter’s activity of its downstream genes to regulate these genes expression, thereby regulating the biological processes of cells [12,13]. As described in previous reports, abnormal expression of FOXF2 was closely related to various tumors development. Dou et al. [14] reported that FOXF2 was down-regulated in hepatocellular carcinoma tissues and cell lines. FOXF2 deficiency could induce EMT in Huh7 cells, which further resulted in the formation of metastasis. Similarly, we detected in this article that low FOXF2 expression was associated with poor outcomes of cervical cancer patients, and that overexpression of FOXF2 inhibited Hela cells proliferation, migration, and invasion *in vitro* and growth *in vivo*. Meanwhile, up-regulated FOXF2 stimulated E-cadherin expression and impaired Vimentin and Snail expression. E-cadherin, Vimentin, and Snail were three EMT-related genes. E-cadherin was a transmembrane glycoprotein distributed on the lateral junction of epithelial cells, which is the molecular basis for mediating cell junctions [15]. Reduction in E-cadherin on the surface of cell membranes disrupted the intercellular connection, thereby resulting in the enhanced invasion and metastasis capacity of tumor cells [16]. Vimentin was considered to be a specific marker of EMT, which was highly expressed in various cancers including prostate cancer, breast cancer, malignant melanoma, lung cancer, and pancreatic cancer [17]. Its expression was positively correlated with tumor growth, invasion, and metastasis [18]. Many studies have shown that down-regulation of Vimentin could significantly inhibit tumor cell invasion and metastasis [19,20]. Furthermore, proliferation of Vimentin-deficient cells was reduced due to the reduction in DNA synthesis [21]. Snail has been shown to be highly expressed in breast cancer, gastric cancer, and colorectal cancer, which plays an important role in promoting tumor metastasis and invasion [22–24]. There was research which also demonstrated that Snail could affect cells biological properties (including tumor cells migration and invasion) by directly interfering with E-cadherin expression in a negatively regulated mechanism [25]. In the present study, E-cadherin, Vimentin, and Snail together regulated Hela cells proliferation, migration, and invasion.

Wnt/β-catenin pathway, one of the classical pathways of the Wnt signaling pathway, played an important role in the development of tumors [26]. Accumulation of β-catenin in the nucleus was an important sign of tumor progression [27,28]. β-catenin was a multifunctional protein which had the dual activity of mediating cell adhesion and signal transduction [29,30]. After accumulation in the nucleus, β-catenin would form a transcription factor complex with the transcription factor TCF/LEF through its C-terminal transcriptional activator binding site, thereby facilitating the transcription of downstream target genes such as *CyclinDl* and *e-mys* [31–33]. In the present study, we observed that FOXF2 could suppress the expression level of β-catenin in the nucleus and target genes expression in the Wnt/β-catenin signaling pathway, such as c-Myc, CyclinDl, MMP9, and Lgr5. As the previous study showed, c-Myc, CyclinDl, MMP9, and Lgr5 were involved in the development of tumors and their overexpression had significant promoting effects on the development of tumors [34,35]. The inhibitory effect of FOXF2 on β-catenin, c-Myc, CyclinDl, MMP9, and Lgr5 will provide an important theoretical basis for the diagnosis and targetted therapy of cervical cancer.

There was a limitation in the present study. The results indicated that overexpression of FOXF2 reduced β-catenin expression level in the nucleus. We speculated that FOXF2 might inhibit β-catenin entry into the nucleus. However, we were currently unable to conduct related researches due to the limitations of laboratory conditions, and this tissue would be the focus of our future research.

In short, this research explored that down-regulation of FOXF2 predicted poor outcomes of cervical cancer patients. Up-regulation of FOXF2 significantly inhibited Hela cells proliferation, migration, and invasion *in vitro* and growth *in vivo*. Overexpressed FOXF2 promoted E-cadherin expression, and suppressed the expression of Vimentin and Snail as well as the expression of target genes in Wnt signaling pathway (including *c-Myc, CyclinDl, MMP9*, and *Lgr5*) and β-catenin in the nucleus. Based on these findings, we speculated that FOXF2 might inhibit the development of cervical cancer by regulating Wnt signaling pathway, which might be a potential target for the diagnosis and treatment of cervical cancer.

## Abbreviations

DAB: diaminobenzidine
DMEM: Dulbecco’s modified Eagle’s medium
hEEC: human endometrial epithelial cell
EMT: epithelial–mesenchymal transition
FOXF2: forkhead box f2
GAPDH: gylceraldehyde-3-phosphate dehydrogenase
OD: optical density
qRT-PCR: quantitative real-time polymerase chain reaction
RIPA: radio immunoprecipitation assay
TBST: tris-buffered saline tween-20
TCF/LEF: T-cell factor/lymphoid enhancer-binding factor
Wnt: wingless-type

## References

[1] Tewari K.S. (2014) Improved survival with bevacizumab in advanced cervical cancer. N. Engl. J. Med. 370, 734–743 10.1056/NEJMoa1309748 24552320 PMC4010094

[2] Liu B. (2016) Seven protective miRNA signatures for prognosis of cervical cancer. Oncotarget 7, 56690–56698 27447860 10.18632/oncotarget.10678 PMC5302945

[3] Yuan L.J. (2015) SPAG5 upregulation predicts poor prognosis in cervical cancer patients and alters sensitivity to taxol treatment via the mTOR signaling pathway. Cell Death Dis. 5, e1247 10.1038/cddis.2014.222 PMC4047857 24853425

[4] Wang H. (2013) Clinicopathological risk factors for recurrence after neoadjuvant chemotherapy and radical hysterectomy in cervical cancer. World J. Surg. Oncol. 11, 301 10.1186/1477-7819-11-301 24266990 PMC4222614

[5] Yun J.A. (2016) Local recurrence after curative resection for rectal carcinoma:The role of surgical resection. Medicine (Baltimore) 95, e3942 10.1097/MD.0000000000003942 27399067 PMC5058796

[6] Kong P.Z. (2016) Decreased expression of FOXF2 as new predictor of poor prognosis in stage I non-small cell lung cancer. Oncotarget 7, 55601–55610 10.18632/oncotarget.10876 27487137 PMC5342439

[7] Kong P.Z. (2013) Decreased FOXF2 mRNA expression indicates early-onset metastasis and poor prognosis for breast cancer patients with histological grade II tumor. PLoS ONE 8, e61591 10.1371/journal.pone.0061591 23620774 PMC3631231

[8] Wang Q.S. (2015) FOXF2 deficiency promotes epithelial-mesenchymal transition and metastasis of basal-like breast cancer. Breast Cancer Res. 17, 30 10.1186/s13058-015-0531-1 25848863 PMC4361145

[9] Chen X. (2017) FOXF2 promoter methylation is associated with prognosis in esophageal squamous cell carcinoma. Tumour Biol. 39, 1010428317692230 28222662 10.1177/1010428317692230

[10] Hirata H. (2013) MicroRNA-182-5p promotes cell invasion and proliferation by down regulating FOXF2, RECK and MTSS1 genes in human prostate cancer. PLoS ONE 8, e55502 10.1371/journal.pone.0055502 23383207 PMC3559583

[11] Zhang Y. (2015) miR-182 promotes cell growth and invasion by targeting forkhead box F2 transcription factor in colorectal cancer. Oncol. Rep. 33, 2592–2598 10.3892/or.2015.3833 25738520

[12] Shi Z (2016) Loss of FOXF2 expression predicts poor prognosis in hepatocellular carcinoma patients. Ann. Surg. Oncol. 23, 211–217 10.1245/s10434-015-4515-2 25824262

[13] Carlsson P. and Mahlapuu M. (2002) Forkhead transcription factors: key players in development and metabolism. Dev. Biol. 250, 1–23 10.1006/dbio.2002.0780 12297093

[14] Dou C. (2017) FOXF2 deficiency promotes hepatocellular carcinoma metastasis by inducing mesenchymal-epithelial transition. Cancer Biomarkers 19, 447–454 10.3233/CBM-170139 28582850 PMC13020746

[15] Cristina I.R. (2016) E-cadherin-160 C/A genotypes and cervical intraepithelial neoplasia. J. BUON 21, 1184–1188 27837621

[16] Lee G., Kim H.J. and Kim H.M. (2016) RhoA-JNK regulates the E-cadherin junctions of human gingival epithelial cells. J. Dent. Res. 95, 284–291 10.1177/0022034515619375 26635280

[17] Satelli A. and Li S. (2011) Vimentin in cancer and its potential as a molecular target for cancer therapy. Cell. Mol. Life Sci. 68, 3033–3046 10.1007/s00018-011-0735-1 21637948 PMC3162105

[18] Satelli A. and Li S. (2011) Vimentin as a potential molecular target in cancer therapy Or Vimentin, an overview and its potential as a molecular target for cancer therapy. Cell. Mol. Life Sci. 68, 3033–3046 10.1007/s00018-011-0735-1 21637948 PMC3162105

[19] Ran L. (2017) CPEB4 promotes cell migration and invasion via upregulating Vimentin expression in breast cancer. Biochem. Biophys. Res. Commun. 489, 135–141 28536077 10.1016/j.bbrc.2017.05.112

[20] Piotrowski-Daspit A.S., Tien J. and Nelson C.M. (2016) Interstitial fluid pressure regulates collective invasion in engineered human breast tumors via Snail, vimentin, and E-cadherin. Integr. Biol. 8, 319–331 10.1039/C5IB00282F PMC4792648 26853861

[21] Herrmann H. and Aebi U. (2004) Intermediate filaments: molecular structure, assembly mechanism, and integration into functionally distinct intracellular scaffolds. Annu. Rev. Biochem. 73, 749–789 10.1146/annurev.biochem.73.011303.073823 15189158

[22] Zhang Y.F. (2016) miR-410-3p suppresses breast cancer progression by targeting Snail. Oncol. Rep., 36, 480–486 10.3892/or.2016.4828 27221455

[23] Cho H.J. (2014) RhoGDI2 promotes epithelial-mesenchymal transition via induction of Snail in gastric cancer cells. Oncotarget 5, 1554–1564 24721928 10.18632/oncotarget.1733 PMC4039231

[24] Sobierajska K. (2016) β-III tubulin modulates the behavior of Snail overexpressed during the epithelial-to-mesenchymal transition in colon cancer cells. Biochim. Biophys. Acta 1863, 2221–2233 10.1016/j.bbamcr.2016.05.008 27188792

[25] Cano A. (2000) The transcription factor snail controls epithelial-mesenchymal transitions by repressing E-cadherin expression. Nat. Cell Biol. 2, 76–83 10.1038/35000025 10655586

[26] Gao L. (2017) Wnt/β-catenin signaling pathway inhibits the proliferation and apoptosis of U87 glioma cells via different mechanisms. PLoS ONE 12, e0181346 10.1371/journal.pone.0181346 28837560 PMC5570310

[27] Thrasivoulou C., Millar M. and Ahmed A. (2013) Activation of intracellular calcium by multiple Wnt ligands and translocation of β-catenin into the nucleus: a convergent model of Wnt/Ca^2+^ and Wnt/β-catenin pathways*. J. Biol. Chem. 288, 35651–35659 10.1074/jbc.M112.437913 24158438 PMC3861617

[28] Jamieson C., Sharma M. and Henderson B.R. (2014) Targeting the β-catenin nuclear transport pathway in cancer. Semin. Cancer Biol. 27, 20–29 10.1016/j.semcancer.2014.04.012 24820952

[29] Fuchs S.Y. (2005) Oncogenic beta-catenin signaling networks in colorectal cancer. Cell Cycle 4, 1522–1539 10.4161/cc.4.11.2129 16258275

[30] Laxmidevi L.B. (2010) Aberrant β-catenin expression in the histologic differentiation of oral squamous cell carcinoma and verrucous carcinoma: an immunohistochemical study. J. Oral Sci. 52, 633–640 10.2334/josnusd.52.633 21206167

[31] Wang L. and Di L.J. (2015) Wnt/β-catenin mediates AICAR effect to increase GATA3 expression and inhibit adipogenesis. J. Biol. Chem. 290, 19458–19468 10.1074/jbc.M115.641332 26109067 PMC4528110

[32] Yu S. (2013) atf4 promotes β-catenin expression and osteoblastic differentiation of bone marrow mesenchymal stem cells. Int. J. Biol Sci. 9, 256–266 10.7150/ijbs.5898 23494915 PMC3596711

[33] Lim K. (2009) Omega-3 polyunsaturated fatty acids inhibit hepatocellular carcinoma cell growth through blocking beta-catenin and cyclooxygenase-2. Mol. Cancer Ther. 8, 3046–3055 10.1158/1535-7163.MCT-09-0551 19887546 PMC2783299

[34] Li Y.J. (2005) β-catenin up-regulates the expression of cyclinD1, c-myc and MMP-7 in human pancreatic cancer: relationships with carcinogenesis and metastasis. World J. Gastroenterol. 11, 2117–2123 10.3748/wjg.v11.i14.2117 15810077 PMC4305780

[35] Yin H. (2017) Overexpression of SOX18 promotes prostate cancer progression via the regulation of TCF1, c-Myc, cyclin D1 and MMP-7. Oncol. Rep. 37, 1045–1051, 10.3892/or.2016.5288 27922675

